# Does the Addition of Zinc Oxide Nanoparticles Improve the Antibacterial Properties of Direct Dental Composite Resins? A Systematic Review

**DOI:** 10.3390/ma14010040

**Published:** 2020-12-24

**Authors:** Divya Arun, Dulanja Adikari Mudiyanselage, Rumana Gulam Mohamed, Michael Liddell, Nur Mohammad Monsur Hassan, Dileep Sharma

**Affiliations:** 1 College of Medicine and Dentistry, James Cook University, 14-88 McGregor Road, Smithfield, QLD 4878, Australia; divya.arun@my.jcu.edu.au (D.A.); dulanja.adikarimudiyaselag@my.jcu.edu.au (D.A.M.); rumana.gulammohamed@my.jcu.edu.au (R.G.M.); 2 College of Science and Engineering, James Cook University, 14-88 McGregor Road, Smithfield, QLD 4878, Australia; michael.liddell@jcu.edu.au; 3 School of Dentistry & Health Sciences, Charles Sturt University Orange Campus, P.O. Box 883, Orange, NSW 2800, Australia; nhassan@csu.edu.au; 4 Australian Institute of Tropical Health and Medicine, James Cook University, Cairns, QLD 4878, Australia; 5 Centre for Molecular Therapeutics, James Cook University, Cairns, QLD 4878, Australia

**Keywords:** antibacterial agents, composite resin, zinc oxide, nanoparticles, zinc oxide nanoparticles, dentistry, dental materials, systematic review

## Abstract

A promising approach to improve the poor antibacterial properties of dental composite resins has been the addition of metal oxide nanoparticles into the resin matrix. This systematic review aimed to determine whether the addition of zinc oxide nanoparticles (ZnO-NPs) improves the antibacterial properties of direct dental composite resins. This review was conducted in accordance with the Preferred Reporting Items for Systematic Reviews and Meta-Analyses (PRISMA) guidelines and registered with the PROSPERO database: CRD42019131383. A systematic literature search was conducted using the following databases: Medline (Ovid), the Cochrane Library, SCOPUS, CINAHL, Web of Science, Trove, Google Scholar, World Cat, and OpenGrey. The initial search retrieved 3178 results, which were then screened against inclusion and exclusion criteria, resulting in a total of four studies that were eligible for qualitative synthesis within this review. All the included studies were in vitro non-randomized post-test design experimental studies. A lack of congruity in the results obtained from these studies that used different tests to evaluate antibacterial activity was evident. Although some studies demonstrated a significant improvement of antibacterial properties in composites containing at least 1% ZnO-NPs (wt %), they are unlikely to present any clear clinical advantage due to the short lifetime of observed antibacterial properties.

## 1. Introduction

Dental caries is a widespread infectious disease, in which the hard tooth structure is demineralized as a result of the acid produced by the bacterial fermentation of carbohydrates. Various direct restorative materials have been used to restore (fill) these carious defects ranging from metallic fillings (silver amalgam, direct gold) to cements (silicates, phosphates, glass-ionomer) and polymer resin/inorganic filler-based composite resins [1,2]. Composite resins are widely used and have predominantly replaced amalgam restorations due to their superior aesthetics and bonding ability in restorative procedures [2,3]. However, some in vitro and in vivo studies have reported that composite resin surfaces, due to its surface roughness, tend to harbor more bacterial plaque when compared with other restorative materials such as silver amalgam, glass ionomer cements (GIC), and also dental hard tissues such as enamel [4,5,6,7]. Other technique-related factors that can significantly affect the longevity of composite resin restorations include the adhesive system and etching agent used [3,8,9]. Composite resin materials are known to undergo polymerization shrinkage at margins, making them susceptible to secondary caries and eventually failure of the restoration [10]. Therefore, the development of dental composites that can resist plaque accumulation and as a result decrease bacterial acid-induced demineralization could increase the longevity of direct dental composite resin restorations [11]. A significant amount of research has been conducted on the incorporation of antibacterial agents into direct dental composite resins. Examples of these agents include fluoride, chlorhexidine, quaternary ammonium, and metal oxide particles/ions such as silver, gold, titanium, and zinc [12,13,14,15,16,17].

One promising approach has been the addition of metal oxide nanoparticles into the resin matrix [18]. As the antibacterial activity of metal oxides is dependent on the total contact surface area, the incorporation of nanoparticulate (1–100 nm) metal oxides allows for superior antibacterial properties, as the surface to volume ratio increases exponentially with decreasing particle size [19,20]. Similarly, silver nanoparticles have been incorporated as antibacterial agents without causing significant detrimental changes to the mechanical properties of dental composites [21]. However, the inclusion of silver causes discoloration of the composite, which is not favorable for aesthetic restorations, restricting its use primarily to restorations on posterior teeth [22].

Traditionally, nanofilled composites are known to allow for desired polishing results and are hence widely used for direct anterior restorations with an acceptable clinical longevity [23,24]. Furthermore, an insoluble, white, or colorless particle with long-lasting antimicrobial properties are the ideal characteristics of an antibacterial filler to be used in a direct dental composite resin [18]. Zinc oxide nanoparticles (ZnO-NPs) have these characteristics, and when incorporated into dental composite resins, they display strong antibacterial properties [19,25]. Therefore, this systematic review was conducted to evaluate the current literature on the addition of zinc oxide nanoparticles and establish if these additives improve the antibacterial properties of direct dental composite resins.

## 2. Materials and Methods

### 2.1. Protocol and Registration

This systematic review was conducted in accordance with the guidelines of the Preferred Reporting Items for Systematic Reviews and Meta-Analyses (PRISMA) statement [26] and was registered with PROSPERO (registration no. CRD42019131383) [27].

### 2.2. Eligibility Criteria

The articles considered for inclusion were in vitro, in vivo, and ex vivo experimental studies. The inclusion criteria were as follows: (a) includes zinc oxide nanoparticles incorporated into direct dental composite resins; (b) investigates the antibacterial properties of direct dental composite resins incorporating zinc oxide nanoparticles; and (c) does not investigate only the synergistic antibacterial effects of multiple agents when zinc oxide nanoparticles are included along with other antibacterial agent(s) in direct dental composite resins.

Exclusion criteria were as follows: (a) articles not published in the English language; (b) articles for which the full text was inaccessible; (c) case series, case reports, conference articles/proceedings, book chapters, theses, dissertations, reviews, ideas, editorials, and opinions.

### 2.3. Information Sources

An electronic search was carried out into the following bibliographic databases and gray literature databases: Medline (Ovid); The Cochrane Library (the Cochrane Database of Systematic Reviews, the Cochrane Central Register of Controlled Trials, and the Cochrane Methodology Register); Scopus; CINAHL; Web of Science; Trove; Google Scholar; World Cat; and OpenGrey. There were no restrictions on publication date, language, or study type in the search.

### 2.4. Focus Question

The following focus question was based on the PICO (Problem, Intervention, Comparison, Outcomes) schema [28] and used to guide the search strategy: “Do direct dental resin composites containing zinc oxide nanoparticles have better antibacterial properties than conventional dental resin composites?”.

### 2.5. Search Strategy

The articles were searched and retrieved using different combinations of medical subject headings (MeSH) terms/subject headings and natural language terms/phrases which in all instances encompassed the following integral concepts of the focus question: (a) antibacterial activity, (b) direct dental composite resin, and (c) zinc oxide nanoparticles. No limits on year of publication were placed for the database searched, and the last search was conducted in May 2020. The combinations and permutations of search terms used were optimized for each database according to the functionality of each database. The following limits determined the optimized search strategy for each database: (a) the number of retrieved articles after completion of the initial search must be less than the maximum number of articles retrievable by the database; (b) search syntax variations for each database; and (c) character limits of the database (Table 1).

### 2.6. Study Selection

The titles and/or abstracts of the articles retrieved were independently screened by two reviewers to identify articles that potentially met the eligibility criteria. Articles that definitively did not meet inclusion criteria and/or fulfilled exclusion criteria at this stage were eliminated. Any disagreements between the two review authors with regard to the eligibility of any particular article were resolved by consultation with a third reviewer until an agreement was reached.

For articles that passed this process, the full-text manuscripts were catalogued and independently assessed by four reviewers for eligibility as per the eligibility criteria.

### 2.7. Data Collection Process

Data from the full-text manuscripts of the articles identified as eligible for the review were extracted independently by two reviewers according to the data items listed in below. Any discrepancies or uncertainties in the data were resolved through discussion with a third review author until an agreement was reached.

### 2.8. Data Items

The data relevant to the research question was extracted from the included studies and tabulated into the following fields for qualitative synthesis:

- Author (year)—presents the author(s) of the article and the year of publication
- Sample—describes the study sample (i.e., type of composite resin)
- Treatment group—describes the treatment group(s), including the average size of zinc oxide nanoparticles used
- Experiment—names the test conducted (N.B: only data for the tests that assessed the antibacterial properties of the ZnO-NP composites were included and tabulated)
- Methodology—describes pertinent information on the corresponding test and the variable(s) measured
- Outcome—describes the outcome(s) of the experiment(s). All outcomes presented are statistically significant unless specified otherwise.

### 2.9. Quality and Risk of Bias in Individual Studies

The quality and risk of bias assessments were performed independently by two review authors using a modified version of the Methodological Index for Non-Randomized Studies (MINORS) tool (Table 2) [29]. Any disagreements or uncertainties were resolved through discussion with a third review author until an agreement was reached.

## 3. Results

### 3.1. Study Selection

**Figure 1:**
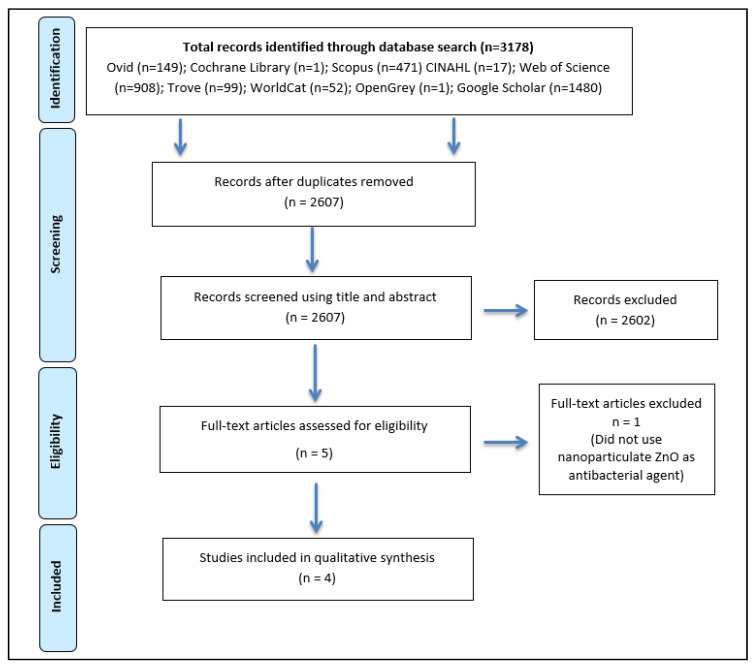
Preferred Reporting Items for Systematic Reviews and Meta-Analyses (PRISMA) flow diagram.

The initial electronic search of the various databases yielded 3178 results. After the removal of duplicates, independent screening of titles and/or abstracts, five studies remained for assessment of eligibility based on full-text review. One study by Shen et al. (2016) was excluded from the review as nanoparticulate zinc oxide was not used in this study [30]. A total of four studies met the all the eligibility criteria and are included in this systematic review (Figure 1).

### 3.2. Study Characteristics

All of the four studies included in this review were non-randomized in vitro experimental studies. The data extracted from the included studies are presented in Table 3.

Three of the studies included in this review investigated the antibacterial properties of ZnO-NP composite resins via a direct contact test where the variable of viable colony-forming units (CFUs) was measured [11,25]. In the studies by Hojati et al. and Kasraei et al., it was reported that the composite resin discs containing at least 1 wt % ZnO-NPs had a statistically significant reduction in the number of CFUs [11,25]. However, Sevinç et al. noted no statistically significant difference in the 1% ZnO-NP composite resins compared to the unmodified control [25]. Nevertheless, all three studies reported a reduction in the number of CFUs with higher concentrations of ZnO-NPs, with the notable exception of the three-species biofilm direct contact test by Sevinç et al. In this test, a three species bacterial inoculum of *S. oralis*, *S. gordonii*, and *A. naeslundii* was used with a 10 wt % ZnO-NP composite resin. The authors found no statistically significant reduction in the number of CFUs between the 10 wt % ZnO-NP composite resin and the control [25].

### 3.3. Quality and Risk of Bias Assessment

**Figure 2:**
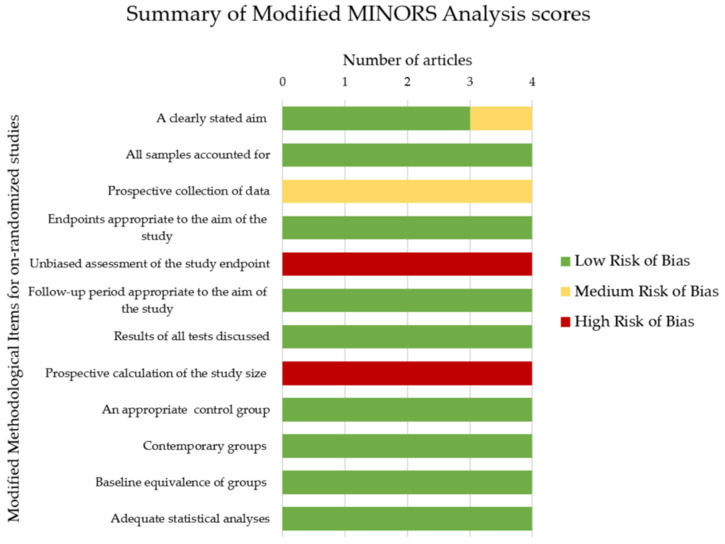
Summary of modified Methodological Index for Non-Randomized Studies (MINORS) analysis scores.

The quality and risk of bias assessments for the four articles included in the review were carried out using the modified version of the Methodological Index for Non-Randomized Studies (MINORS) tool [29] and are summarized in Figure 2 and Table 4. The primary sources of bias in the four articles are the absence of operator blinding, the lack of publication of the study protocol prior to conducting the study, and the absence of a prospective calculation of the study sample size required. Hence, all studies scored 1 for item three and 0 for items five and eight in the modified MINORS tool. Overall, all articles scored between 18 and 19 points in total, hence, presenting with a moderate risk of bias.

## 4. Discussion

Much research has been carried out on the addition of antibacterial agents into direct dental composite resins to improve their antibacterial properties [12,13,14,15,16,17]. A promising approach to achieve this has been the incorporation of metal oxide nanoparticles, such as zinc oxide, as a filler into the resin matrix. A Cochrane review, updated in 2013, by Pereira-Cenci et al. attempted to assess the state of the research on antibacterial agents incorporated into dental composite resins, limiting the scope of their review to only randomized controlled trials. They were unable to find any studies that met this criterion [33]. However, the present systematic review did not place any limitations on study design and focused exclusively on the addition of zinc oxide nanoparticles as an antibacterial agent, and hence yielded four studies that were eligible for inclusion in this review. Nevertheless, similar to the findings of Pereira-Cenci et al., no randomized controlled trials were found; all of the studies included in this review are in vitro non-randomized post-test design experimental studies. The rationale for this systematic review was to evaluate the evidence that the addition of zinc oxide nanoparticles to composite restorative resins improves the antibacterial properties of direct dental composite resins.

Overall, there was a lack of congruity in the findings of the studies between the different tests/methodologies used to evaluate the antibacterial activity of ZnO-NP composite resins at lower weight percentage ZnO-NP loadings. Two studies included in this systematic review reported a significant improvement of antibacterial activity in composite resins containing at least 1 wt % ZnO-NPs, compared to the controls, in short-term direct contact tests utilizing a single species biofilm [19,31]; another study had similar findings in composite resins containing at least 2 wt % ZnO-NPs [25].

In all of the three studies that assessed samples under electron microscopy, a qualitative reduction in the number of bacterial colonies was found with increasing ZnO-NP concentrations; however, the concentrations at which these changes were observed differed considerably amongst the three studies—1 wt % being the lowest effective loading depending on the study [19,25,32]. One of the studies measured biofilm metabolic activity and lactic acid formation, and it came to the conclusion that composites containing at least 1 wt % ZnO-NP reduce biofilm metabolic activity, but that a decrease in lactic acid production is only seen at concentrations of 5 wt % ZnO-NPs or greater [32].

Three studies that investigated the antibacterial properties used a direct contact test followed by viable colony-forming units (CFUs) measurement reported variable results, specifically in relation to composite resins containing a lower concentration of ZnO-NPs [19,25,31]. Notable reasons were differences in the direct contact test used; only Sevinç et al. used an *S. sobrinus* biofilm, whereas the other two studies used a *S. mutans* or *Lactobacillus* biofilm [25]. In addition, the age of the biofilms used differed among the three studies, with the *S. sobrinus* biofilm of Sevinç et al. being at least 48 h more mature than those used in the other two studies.

In the literature, some studies suggest that ZnO-NP composites may have little antibacterial effect against multi-species biofilms [25]. In regard to the findings of the three species biofilm direct contact test reported by Sevinç et al., the differences may have been due to the stronger antibacterial resistance of multi-species bacterial biofilms or the species of bacterium themselves [34]. This may suggest that in a clinical situation, ZnO-NP composite resins may not provide any benefit over regular dental composites.

Based on experiments that attempted to simulate the aging of composite restorations, the literature suggests that the antibacterial effect of the ZnO-NP composites may not be long-lasting [19,25]. Of the studies included in this review, only Sevinç et al. and Hojati et al. attempted to simulate the effect of restoration aging on the antibacterial properties of the composite resin samples [19,25]. The methodologies used by the two studies were considerably different from one another; Sevinç et al. aged their composite resin samples by subjecting the samples to three cycles of 72 h of biofilm growth, whereas Hojati et al. aged their samples by subjecting them to a phosphate-buffered saline bath for 2–28 days [19,25]. Sevinç et al. found no diminishing of antibacterial activity in the 10 wt % ZnO-NP composite resins after the first and second cycle of growth but did note a statistically significant reduction in antibacterial activity by way of increased CFUs in the third cycle of growth [25]. Nevertheless, in all three cycles, the number of CFUs was less than the number of CFUs detected for the unmodified control. Hojati et al. found that in composite resin discs aged for 48 h, there was a reduction in the number of CFUs in all the ZnO-NP composites compared to the control [19]. However, in their samples, which were aged for 1 week and 4 weeks, no statistically significant difference in the number of CFUs was detected between any of the ZnO-NP composites and the control [19,25]. This is not surprising, as the use of a PBS buffer solution would have rapidly degraded the ZnO-NP due to the known sensitivity of ZnO-NP to phosphate [35]. Agar diffusion tests performed by Sevinç et al. and Hojati et al. did not reveal a zone of inhibition around any of the composite samples, suggesting that there is no peripheral antibacterial effect with the ZnO-NP composite resins due to the insolubility of the ZnO-NPs [19,25]. Taken together, these results suggest that in a clinical situation, in which a composite resin restoration will be exposed to the varying chemistry of the oral cavity for many years, the antibacterial effectiveness of ZnO-NP in ZnO-NP composite resins may be negligible.

Three of the four studies included in this review visualized their samples of bacterial colonization of composite resin under a scanning electron microscope (SEM) and/or a confocal laser scanning microscope (CLSM) [19,25,32]. Sevinç et al. found that *S. sobrinus* attachment and biofilm coverage and density were all qualitatively lower on the 10 wt % ZnO-NP composites compared to unmodified control composites in both SEM and CLSM [25]. Hojati et al. observed that in samples aged for either 1 h or 24 h, there was a qualitatively lower number of and smaller bacterial colonies in composite resins containing higher concentration of ZnO-NPs (3, 4 and 5 wt %) compared to the composite resins containing a lower concentration of ZnO-NPs (0, 1, and 2 wt %) under the SEM [19]. Finally, Brandão et al. noted a decrease in the number of colonies in the 1, 2, 5, and 10 wt % ZnO-NP composites compared to control under the SEM, indicating a significant improvement in antibacterial effectiveness as the ZnO-NP concentration increased [32]. Nevertheless, the results of this image analysis should be interpreted with caution, as there is a high risk of bias in sample selection, image selection, and interpretation of SEM or CLSM images by all three studies [19,25,32].

In addition to the above-mentioned tests, two of the studies performed tests unique to their respective studies [25,32]. These included the kinetic measurement of bacterial growth test by Sevinç et al., and the metabolic activity assay (MTT) and lactic acid production tests by Brandão et al. [25,32]. In the kinetic measurement of bacterial growth test, it was noted that 1, 5, and 10 wt % ZnO-NP composites showed a reduction in absorbance measurements compared to the positive control and hence improved the antibacterial activity against planktonic cultures of *S. sobrinus*; no statistically significant difference was found between these composites [25]. In the metabolic activity assay, it was found that the 1, 2, 5, and 10 wt % ZnO-NP composites decreased the metabolic activity of the *S. mutans* biofilm compared to the control. The 0.5 wt % ZnO-NP composite did not show a statistically significant difference compared to the control [32]. Finally, in the lactic acid production test, only the 5 and 10 wt % ZnO-NP composites reduced the production of lactic acid by the *S. mutans* biofilm [32]. This suggests that with low-concentration ZnO-NP composite resins that are required for mechanical stability clinically, there will be little effect on secondary caries prevention, as at low wt % ZnO, there is insufficient inhibition of the formation of lactic acid, which causes tooth demineralization.

Two of the studies included in this review also assessed changes in the mechanical and physiochemical properties of the composite resins as a result of the addition of ZnO-NP into the resin matrix. Hojati et al. concluded that the addition of up to 1 wt % ZnO-NPs into composite resins does not adversely affect the mechanical properties of the composite [19]. No adverse effects due to the addition of the ZnO-NP on the degree of conversion, flexural strength, compressive strength, and bond strength were found. However, they noted that as ZnO-NP concentrations increase, the depth of cure decreased significantly, finding that composite resins containing 5 wt % ZnO-NP had half the depth of cure of the unmodified control group [19]. Brandão et al. found that for 2–5 wt % ZnO-NP composite resins, there were no adverse effects on degree of conversion, flexural strength, elastic modulus, water sorption, and water solubility [32]. However, they found that microhardness and translucency were negatively affected by the addition of ZnO-NPs to the resin matrix of dental composites above 1% wt % [32].

The studies included in this systematic review display three primary limitations; study design, short term evaluation of antibacterial activity, and limited bacterial species against which the antibacterial effects were evaluated. All the studies included in this review are in vitro studies; therefore, the results may not translate to clinically relevant conclusions. Conditions in an oral cavity differ substantially from the in vitro conditions used in the included studies. The biochemical nature of the oral cavity is constantly changing as it is being flushed with saliva, food, and fluid drinks. This dynamic chemical environment has the potential to alter the results/conclusions drawn from these studies of the antibacterial agents in dental composites in controlled in vitro settings [36]. Furthermore, none of the studies had any operator blinding, potentially leading to bias in the interpretation of data, especially in tests where subjective interpretation was required, such as the scanning electron microscopy and confocal laser scanning microscopy.

Most of the tests used to assess antibacterial activity were short-term (1–3 days). Long-term studies of the antibacterial effect of ZnO-NP are yet to be conducted. A short-term exposure of zinc nanoparticle incorporated composite against bacteria does not represent how well the restorations will perform in the oral cavity over an extended period of time. Around 60% of composite resin restorations are expected to last for more than 10 years, if sound restorative principles and techniques are employed during placement [8]. Therefore, a long-term study of ZnO-NP dental composites in vivo is required to provide a more definitive answer as to the efficacy of ZnO-NP as antibacterial agents in restorations in the oral cavity. This would only be warranted once ZnO-NP composites are developed that clearly demonstrate long-term (>1 week) anti-microbial activity in in vitro tests, as this has yet to be established.

Finally, most of the studies included in this review used single species biofilms to test the antibacterial activity of ZnO-NP dental composites except for Sevinç et al., who used a three-species biofilm for a single test, and in this test, they found no statistically significant difference between the ZnO-NP composites compared to the control [25]. Due to the symbiotic activity of oral biofilms, testing against a wide range of oral species for an extended period may yield results that are more relevant to a clinical situation [34].

## 5. Conclusions

There is a lack of congruity in the data from these studies due to the variation in assessment methods used to evaluate the antibacterial activity of ZnO-NP direct dental composite resins. Some tests such as the single species direct contact tests, metabolic assay tests, and visualization under electron microscopy demonstrated a significant improvement of antibacterial properties in composites containing at least 1 or 2 wt % ZnO-NPs compared to controls. Other tests such as aged direct contact test, multi-species direct contact tests, and the lactic acid production test showed little to no difference compared to the controls, particularly at lower ZnO-NP concentrations, or over extended periods of time. Current evidence suggests that ZnO-NP composites are unlikely to present any clear clinical advantage due to the short lifetime of observed anti-bacterial properties, and the poor results against multi-species biofilms in the in vitro studies examined in this systematic review. Further research is warranted in developing ZnO-NP with enhanced long-term bioactivity and enhanced antibacterial efficacy against multi-species biofilms. Efforts to improve and develop standardized study designs to mimic the oral environment in vivo and methods for the optimization of materials including the ZnO-NP are paramount.

## Figures and Tables

**Table 1:** Search strategy for databases searched.

Database(s)	MeSH Terms/Subject Headings and/or Keyword Search
Medline (Ovid)	exp Antibacterial Agents/ (bacteriostatic or bacteristatic or bactericidal* or bacteriocidal* or anti-bacterial or antibacterial or bacteriocidal or anti-microbial* or antimicrobial*).mp. 1 or 2 exp Composite Resins/ (composite*).mp. 4 or 5 exp Zinc Oxide/ (“zinc oxide” or zinc-oxide or ZnO or ZnO-NP or Nano-ZnO or “Nano ZnO” or nano-zinc-oxide or “nano zinc oxide”).mp. 7 or 8 exp Nanoparticles/ (nanoparticle* or nanoparticle*).mp. 10 and 11 3 and 6 and 9 and 12
Cochrane Library	(antibacterial OR anti-bacterial OR bactericidal OR bacteriocidal OR bacteriostatic OR bacteriostatic OR anti-microbial OR antimicrobial) in All Text AND composite in All Text AND (Zinc-Oxide OR “Zinc Oxide” OR ZnO OR ZnO-NP OR Nano-ZnO OR “Nano zinc oxide” OR Nano-zinc-oxide) in All Text AND (nanoparticle OR nano-particle) in All Text—(Word variation have been searched)
SCOPUS	(TITLE-ABS-KEY (zno OR “zinc oxide” OR zinc-oxide OR zno-np OR “Nano ZnO” OR nano-zno OR “Nano zinc oxide” OR nano-zinc-oxide) AND TITLE-ABS-KEY (composite) AND TITLE-ABS-KEY (antibacterial OR bacteriostatic OR bacteriocidal OR anti-microbial* OR antimicrobial* OR anti-bacterial* OR bactericidal* OR bacteristatic) AND TITLE-ABS-KEY (nanoparticle* OR nano-particle*))
CINAHL	(ZnO OR “zinc oxide” OR Zinc-Oxide OR ZnO-NP OR “Nano ZnO OR Nano-ZnO OR “Nano zinc oxide” OR Nano-zinc-oxide) AND composite* AND ((Antibacterial OR bacteriostatic OR bacteriocidal OR anti-microbial* OR antimicrobial* OR Anti-bacterial OR Bactericidal* OR bacteristatic)) AND ((nanoparticle* OR nano-particle))
Web of Science	TS = (Antibacterial OR Anti-bacterial OR Bactericidal* OR Bacteriostatic OR Bacteristatic OR Anti-microbial* OR Antimicrobial*) TS = (Composite*) TS = (Zinc-Oxide OR “Zinc Oxide” OR ZnO OR ZnO-NP OR “Nano ZnO” OR Nano-ZnO OR “Nano zinc oxide” OR Nano-zinc-oxide) TS = (Nanoparticle* OR Nano-particle*) #4 AND #3 AND #2 AND #1
Trove	(ZnO OR “zinc oxide” OR Zinc-Oxide OR ZnO-NP OR “Nano ZnO OR Nano-ZnO OR “Nano zinc oxide” OR Nano-zinc-oxide) AND composite* AND ((Antibacterial OR bacteriostatic OR bacteriocidal OR anti-microbial* OR antimicrobial* OR Anti-bacterial OR Bactericidal* OR bacteristatic)) AND ((nanoparticle* OR nano-particle)) N.B. Search was carried out in the Journals, articles and data sets tab
Google Scholar	Dental resin composite antibacterial inhibit biofilm nanoparticle “zinc oxide”
World Cat	Dental resin composite antibacterial nanoparticle “zinc oxide”
OpenGrey	(ZnO OR “zinc oxide” OR Zinc-Oxide OR ZnO-NP OR “Nano ZnO OR Nano-ZnO OR “Nano zinc oxide” OR Nano-zinc-oxide) AND composite* AND ((Antibacterial OR bacteriostatic OR bacteriocidal OR anti-microbial* OR antimicrobial* OR Anti-bacterial OR Bactericidal* OR bacteristatic)) AND ((nanoparticle* OR nano-particle))

**Table 2:** Modified Methodological Index for Non-Randomized Studies (MINORS) tool.

Methodological Items for Non-Randomized Studies (Modified Version)	Score *
A clearly stated aim: the question addressed should be precise and relevant in the light of available literature.	
All samples accounted for: all prepared samples should be accounted for and used as specified in the methods.	
Prospective collection of data: data were collected according to a protocol established before the beginning of the study.	
Endpoints appropriate to the aim of the study: unambiguous explanation of the criteria used to evaluate the main outcome, which should be in accordance with the question addressed by the study. In addition, the endpoints should be assessed on an intention-to-treat basis.	
Unbiased assessment of the study endpoint: there should be a blinding of test operators. In addition, operator variation in measurement should be taken into account where appropriate.	
Follow-up period appropriate to the aim of the study: the follow-up should be sufficiently long to allow the assessment of the main endpoint and possible adverse events.	
Results of all tests discussed: results of all tests done should be presented and discussed.	
Prospective calculation of the study size: information of the size of detectable difference of interest with a calculation of 95% confidence interval, according to the expected incidence of the outcome event, and information about the level for statistical significance and estimates of power when comparing the outcomes.	
An appropriate control group: an appropriate control group must be present.	
Contemporary groups: control and treatment groups should be managed during the same time period using the same batch of antibacterial strains/solutions (no historical comparison).	
Baseline equivalence of groups: control samples must be created using the same procedure as non-control groups, except for steps required for addition of antibacterial agent(s). Absence of confounding factors that could bias the interpretation of the results.	
Adequate statistical analyses: whether the statistics were in accordance with the type of study with calculation of confidence intervals or relative risk.	

* The items were scored 0 (not reported—high risk of bias), 1 (reported but inadequate—medium risk of bias) or 2 (reported and adequate—low risk of bias). The global ideal score being 16 for non-comparative studies and 24 for comparative studies.

**Table 3:** Summary of studies included in this systematic review.

Author (Year)	Sample	Treatment Group(s)	Experiment	Quantification (Variables)	Outcome
Sevinç et al. (2010) [25]	Microhybrid composite resin (AElite All-purpose body) Nanofilled composite resin (Filtek Supreme Plus Universal Restorative)	1, 5, and 10 wt % uncoated ZnO-NPs (40–100 nm) in microhybrid composite resin 1 wt % polar coated ZnO-NPs (40–100 nm) in microhybrid composite resin 1 wt % non-polar coated ZnO-NPs (40–100 nm) in microhybrid composite resin 10 wt % ZnO-NPs (40–100 nm) in nanofilled composite resin	Direct contact test	Viable colony-forming units (CFUs) after 48 h. *S. sobrinus* biofilm.	Reduced CFUs in 5 and 10 wt % ZnO-NP composites compared to controls.
			Aged direct contact test	Viable CFUs after each cycle. Three cycles of 72 h growth of *S. sobrinus* biofilms.	Reduced biofilm growth in the 3rd cycle for 10 wt % ZnO-NP composite compared to the 3rd cycle of the unmodified composite resin.
			Three species biofilm direct contact test	Viable CFUs after 72 h of biofilm growth. Bacterial culture of *S. oralis*, *S. gordonii*, and *A. naeslundii* was used.	No statistically significant reduction in CFUs between 10 wt % ZnO-NP composite resin and controls.
			Scanning electron microscopy (SEM) and confocal laser scanning microscopy (CLSM) of direct contact test	Qualitative description of composite discs via scanning electron microscopy and confocal laser microscopy. Age of *S. sobrinus* biofilm was 24 h.	*S. Sobrinus* attachment and biofilm coverage was qualitatively lower on the 10 wt % ZnO-NP composites compared to unmodified control composites in both SEM and CLSM. Both SEM and CLSM visualization revealed qualitatively less dense biofilm formation and greater space between microbes in the 10 wt % ZnO-NP composite compared to the control.
			Kinetic measurement of bacterial growth	Well suspension culture plates were coated with composite resins and inoculated with bacterial culture for 6 h. The per hour absorbance measurements were recorded using a plate reader (490 nm at 1 h intervals for 12 h).	There was no statistically significant difference between the 1, 5, and 10 wt % ZnO-NP composites at the 14th h. However, all of these composites showed a reduction in absorbance measurements compared to the control composites.
			Agar diffusion test	The inhibition zones around the composite discs were measured after 48 h incubation of bacteria inoculated BHI agar plates.	There was no zone of inhibition around either the ZnO-NP composites or the unmodified composites. In addition, bacterial colonies were observed on the bottom surfaces of these composites.
Hojati et al. (2013) [19]	Flowable composite resin (Heliomolar Flow)	1, 2, 3, 4 and 5 wt % ZnO-NPs (20 nm) in flowable composite resin	Direct contact test	Visual counting of number of viable CFUs after 24 h incubation. Age of the *S. Mutans* biofilms were 3, 6, 12, and 24 h.	There was a reduction in the number of CFUs with an increasing percentage of ZnO-NPs in all of the ZnO-NP composites compared to control. The 4 and 5 wt % ZnO-NP composites completely inhibited bacterial colony formation.
			Aged direct contact test	Visual counting of number of viable CFUs after 3, 6, 12, and 24 h. All samples were incubated for 24 h. All samples were pre-aged with phosphate-buffered saline (PBS) for 48 h, 1 week, and 4 weeks.	In 48 h aged samples, there was a reduction in CFUs in 1, 2, 3, 4, and 5 wt % ZnO-NP composites compared to the control. However, there was no statistically significant difference between the 1, 2, 3, 4, and 5 wt % ZnO-NP composites. In the 1-week and 4-week aged samples, there was no statistically significant difference between the 1, 2, 3, 4, and 5 wt % ZnO-NP composites and the control.
			Agar diffusion test	The inhibition zones around the composite discs were measured after 24 h incubation of bacteria inoculated BHI agar plates.	There were no inhibition zones around any of the composite resin discs containing ZnO-NPs or the control composite resin discs.
			Scanning electron microscopy (SEM) of direct contact test	Qualitative description of visualized samples of composite discs via SEM. Age of *S. Mutans* biofilms were 1 h and 24 h.	For 1 h incubated samples, there was a qualitatively lower number of and smaller bacterial colonies in composite resins containing higher concentration of ZnO-NPs (3, 4 and 5 wt %) compared to the composite resins containing a lower concentration of ZnO-NPs (0, 1, and 2 wt %). The results were similar for samples incubated for 24 h.
Kasraei et al. (2014) [31]	Flowable composite resin (Opallis)	1 wt % ZnO-NPs (50 nm) in flowable composite resin	Direct contact test	Visual counting of number of viable CFUs after 48 h. Age of *S. Mutans* or *Lactobacillus* biofilms were 12 h for both.	There was a reduction in CFUs of in both the *S. mutans* and *Lactobacillus* groups, for the 1% ZnO-NP composites compared to control.
Brandão et al. (2017) [32]	Model composite resin prepared by researchers (BisGMA 70 wt % and TEGDMA 30 wt %; 70% barium borosilicate glass filler)	0.5, 1, 2, 5, and 10 wt % ZnO-NPs (40–100 nm) in model composite resin.	Evaluation of metabolic activity via an MTT [3-(4,5-dimethylthiazol-2-yl)-2,5-diphenyltetrazolium bromide] assay	Based on the enzymatic reduction of yellow tetrazolium into purple formazan. Absorbance values of samples were recorded using a microplate reader (540 nm). Age of *S. Mutans* biofilm was 72 h.	One, 2, 5, and 10 wt % ZnO-NP composites decreased the metabolic activity of the *S. mutans* biofilm compared to the control. The 0.5 wt % ZnO-NP composite did not show a statistically significant difference compared to the control.
			Lactic acid production	Lactic acid analysis of 3 h incubated samples via the use of a lactate dehydrogenase (LDH) reaction (measured in µM). Age of *S. mutans* biofilm was 72 h.	Only 5 and 10 wt % ZnO-NP composites reduced the production of lactic acid by the *S. mutans* biofilm.
			Scanning electron microscopy (SEM) of direct contact test	Qualitative description of visualized samples of composite discs via SEM (5000× magnification).	A qualitative decrease in the number of bacterial colonies was noted in the 1, 2, 5, and 10 wt % ZnO-NP composites compared to control. There was a significant decrease in the number of colonies as ZnO-NP concentration increased.

**Table 4:** Modified MINORS analysis scores for each item.

Study	MINORS Item												Total Score
	1	2	3	4	5	6	7	8	9	10	11	12	
Sevinç et al. (2010)	1	2	1	2	0	2	2	0	2	2	2	2	18
Hojati et al. (2013)	2	2	1	2	0	2	2	0	2	2	2	2	19
Kasraei et al. (2014)	2	2	1	2	0	2	2	0	2	2	2	2	19
Brandão et al. (2017)	2	2	1	2	0	2	2	0	2	2	2	2	19

## Data Availability

Data sharing not applicable. No new data were created or analyzed in this study.
